# Age related grid-wise spatial analysis of choroidal parameters in well characterised healthy population

**DOI:** 10.1038/s41598-024-76844-6

**Published:** 2024-11-04

**Authors:** Meenakshi Kumar, Matt Trinh, Angela Zhang, Xin Wei, Rupesh Agrawal, Lisa Nivison-Smith

**Affiliations:** 1 School of Optometry and Vision Science, University of New South Wales, Sydney, Australia; 2 Centre for Eye Health, University of New South Wales, Sydney, 2052 Australia; 3 National Healthcare Group Eye Institute, Tan Tock Seng Hospital, Singapore, Singapore; 4 Lee Kong Chian School of Medicine, Nanyang Technological University, Singapore, 308433 Singapore; 5 Singapore Eye Research Institute and Singapore National Eye Centre, Singapore, Singapore; 6 Duke NUS Medical School, Singapore, Singapore

**Keywords:** Choroidal vascularity index, Aging, Spatial changes, Choroidal angioarchitecture, Biomarkers, Health care, Medical research

## Abstract

Choroidal vascularity index (CVI) is an image-based metric applied to assess the choroid in disease but poorly explored in natural aging. In this retrospective cohort study, we assessed a single eye of 309 healthy individuals from 20 to 70 + years, and quantified spatial topography of total choroidal area (TCA), luminal area (LA), stromal area (SA) and CVI (LA: TCA) in cluster and standard retinal templates using a semi-automated approach. CVI was found to be greatest in the central vs. periphery (CVI_central_ 67.08% [66.85–67.73] vs. CVI_periphery_65.68% [65.32–66.01], *p* < 0.01). In this study, CVI also showed no significant change with age or physiological factors. Area parameters demonstrated greater magnitude in the central versus periphery but with a superior bias: TCA_superior_ 0.227mm^2^ [0.223–0.233] vs. TCA_inferior_ 0.207mm^2^ [0.201–0.213], *p* < 0.01.They also showed a linear decline with age (TCA:-0.00112 to -0.00169 mm^2^/year; LA: -0.00074 to -0.00112 mm^2^/year; SA: -0.00039 to -0.00056 mm^2^/year, *p* < 0.0001), with a higher rate of decline inferiorly (*p* < 0.001). This study demonstrates that CVI exhibits specific spatial differences across macula, independent of age. It further establishes a comprehensive, normative database of CVI and other choroidal angioarchitecture metrics which is a valuable resource for distinguishing pathological changes from normal age-related variations in future assessments of choroid-involving diseases.

**Supplementary Information:**

The online version contains supplementary material available at 10.1038/s41598-024-76844-6.

## Introduction

The choroidal vascularity index (CVI) is an image-based metric which describes contributions of the choroidal lumen and stroma to the overall choroidal angioarchitecture^1,2,3,4^. CVI has been used to accurately describe choroidal changes in many retinal diseases such as age-related macular degeneration, polypoidal choroidal vasculopathy, diabetic retinopathy, central serious chorioretinopathy, heredomacular degeneration, uveitis and Vogt-Koyanagi-Harada disease^4^. CVI has also been shown to be superior to other choroidal metrics, whereby it is sensitive to subtle changes in the early stages of disease that otherwise are less detectable using choroidal thickness^5,6^.

The choroid demonstrates several variations in angioarchitecture due to normal physiological factors such as age, refractive error, gender, ethnic differences, axial length and spatial location such macular vs. mid- and far periphery. Understanding these normal changes are important to accurately differentiate from disease. Normative descriptions of CVI, however, have been limited and conflicting. For example, several studies have reported CVI to decreases with normal aging^7^, while others have found no correlations between CVI and age^4,8,9,10,11^. Similarly, there are mixed correlations relating axial length and CVI^9,10,11,12,13,14,15,16,17^. These conflicts may arise from limitation in study design, such as not accounting for mixed ethnic groups and methodological shortcomings such as not accounting for refractive error or use of different methods for CVI calculation.

Spatial variations in CVI have been demonstrated in few studies, though this metric has been typically assessed across rudimentary delineations of space, such as coarse wide-ranging eccentricities or the Early Treatment for Diabetic Retinopathy Study (ETDRS) sectors^7,18^. These arbitrary spatial configurations may potentially make inappropriate assumptions of the choroid by assuming perfect symmetry across nine uneven sectors and introduce a statistical bias due to the nature of aggregation^19^. Thus, a comprehensive characterisation of CVI in a well-defined population using appropriate spatial templates is needed to elicit the natural topography without prior assumptions.

Our group has recently developed a grid-wise spatial analysis of optical coherence tomography (OCT) images and shown that this approach can describe detailed spatial topography of individual retinal layers based on thickness that closely aligned to descriptions^20,21,22^and showed less variability than other spatial analyses performed with other templates such as the ETDRS sectors^23^. Additionally, this approach has shown superior detection of retinal thickness differences in Age related macular degeneration and glaucoma versus normal eyes, and visual field defects in age related macular degeneration, relative to traditional methods^24^. This analysis has also been applied to OCT angiography images to quantify spatial patterns of vessel perfusion in normal and disease eyes but to our knowledge, it has yet to be applied to choroidal parameters such as CVI accessible from OCT.

Hence, the aim of this study was to characterise the normal physiological and spatial variations of the CVI and other choroidal parameters using our grid-wise spatial OCT analysis in a large, well-characterised normative population ranging across six decades of life. Finally, we further explore the equivocal relationship between physiological and ocular factors with CVI and Choroidal parameters, generating age-based regression functions for estimation in future studies.

## Methods

### Study population

All participant data was obtained from normative populations previously identified from retrospective analysis of consecutive records from the Centre for Eye Health (CFEH) Sydney, Australia from June 2012 to July 2022^7,20,21,23^. CFEH is a referral-only clinic providing advanced diagnostic eye testing/disease management by specially trained optometrists and ophthalmologists^25^. This study included healthy volunteers and patients who were referred to CFEH and found to have no evidence of any posterior segment diseases based on ocular examination including OCT and mydriatic fundus photography and no systemic vascular related disease like Hypertension, Diabetes mellitus or Cardiovascular abnormalities or mental/cognitive impairment based on self-reported medical history. Inclusion and exclusion criteria were described elsewhere^7^. Briefly, participants were included across a range of 20 to 83 years old and consisted of a minimum of 36 participants per decade group and were excluded if they had a best corrected visual acuity (BCVA) less than 6/9, intraocular pressure (IOP) greater than 21mmHg or had a current or previous history of smoking. To minimise the effect of ocular magnification on scaling^26^, the range of refractive error (RE) was ± 3D in < 40 years and ± 6D for > 40 years to account for variation secondary to age related changes. The study was approved by the Biomedical Human Research Ethics Advisory Panel of the University of New South Wales (HC200833). All participants gave informed, written consent to have their deidentified data used for research purposes in accordance with the Declaration of Helsinki.

### Image extraction and measurement

Spectralis SD-OCT macular cube scans consisting of 61 B scans (scan length: 8.6 mm, scan depth: 1.8 mm, resolution of 3.9 microns) covering an area of 30° X 25°. (Heidelberg Engineering, Heidelberg, Germany) of a single, randomly selected eye from each participant was extracted. If a participant had multiple scans in their file, the latest macular cube scan was used. All scans with less than 15db quality score or poor visibility of choroid due to opacities /shadowing were excluded. If three or more B-scans of the macular cube were to be excluded from a participant for quality reasons, then the participant was excluded from this study. As such no participants were eliminated for this criteria.

**Figure 1:**
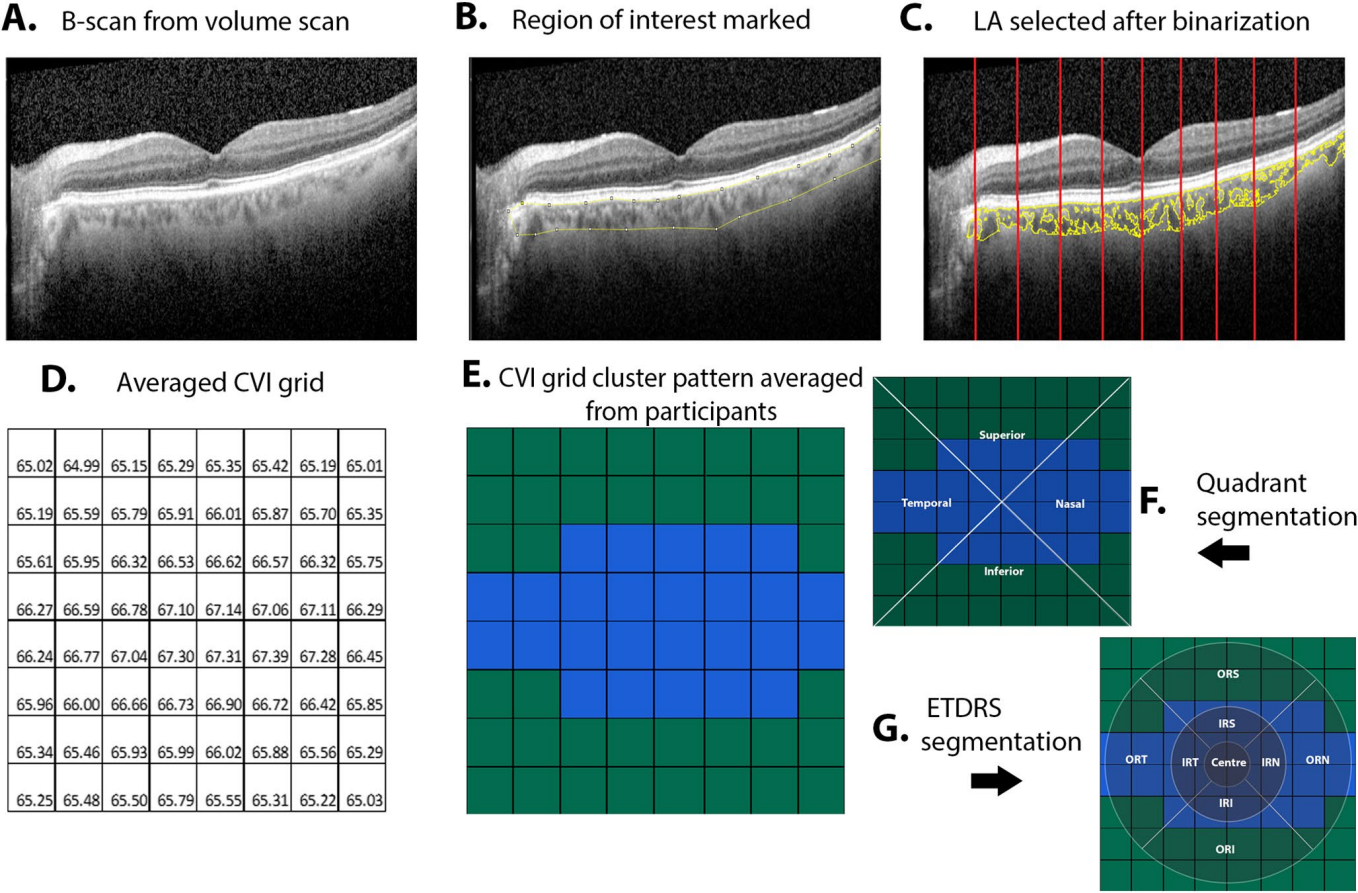
(**A**) Individual B scan extracted from volume scan and (**B**) region of interest representing the total choroidal area (TCA) is marked. (**C**) The luminal area (LA) and stromal areas are then identified as black and white pixels respectively within the ROI from a binarized form of the image (where binarization involves converting all image pixels to black or white only based on a threshold pixel value). The segmentation borders for each grid column is indicated by the red lines. (**D**) CVI is calculated as the ratio of LA: TCA for individual segments in the OCT B scan and then repeated for all B scans of the volume scan before grouping and averaging to generate each of the 64 grid values. (**E**) These values are averaged across participants to form average cluster for this population. (**F**) The grid split into quadrant template (**G**) and ETDRS template where IRS- Inner ring superior, IRN- Inner ring nasal, IRI- Inner ring inferior, IRT-Inner ring temporal, ORS-Outer ring superior, ORN-Outer ring nasal, ORI-Outer ring inferior, ORT-Outer ring temporal. Significance level *p < 0.05, **p < 0.01, *** *p* < 0.001 ,****p < 0.0001.

All B-scans within the posterior segment analysis of the Spectralis in-built software, an 8 × 8 grid-based analysis of the macula (Fig. 1A), were annotated to align with the 8 grid segments. Annotated B scans were then analysed for choroidal parameters as described by Agrawal et al^3^. using the purpose-built platform for automated choroidal image analysis (www.ocularimaging.net/home). Specifically, deidentified B-scans images of participants were uploaded to the platform, then the choroidal region of interest (ROI) was marked from the upper border of the retinal pigment epithelium to the lower border of the choroido-scleral junction (Fig. 1B). Thirty images were annotated by two graders (MK, AZ) who were masked to each other’s ROI markings to validate the annotation protocol (ICC = 0.7, *p* = 0.001) before all remaining images were annotated by a single grader (MK). The binarized image (an image where all pixels are converted to white (pixel value 255) and black pixels (pixel value 0) based on whether they exceed or fall below a threshold pixel value respectively) with ROI was then separated into 8 grid-segments based on the vertical annotations in the B-scan .The luminal area (LA), stromal area (SA) and total choroidal area (TCA) was calculated as the number of black pixels, white pixels, and total pixels, respectively (Fig. 1C), within each grid segment of the ROI. Values were converted from pixel^2^ to mm^2^ using image specifications (X = 85 pixel/mm and Z = 255 pixel/mm). CVI was calculated as LA/TCA (unitless) and converted to a percentage. Choroidal parameters from individual B-scans (Fig. 1C) were then grouped and averaged (Fig. 1D) to generate an 8 × 8 grid that mirrored the presentation of thickness values in the posterior segment analysis of Spectralis software.

### Spatial analysis

8 × 8 grid data for each choroidal parameter was assessed by three forms of spatial analyses. Firstly, data was subjected to Two-step clustering using Euclidean distance in SPSS (version 28.0; IBM Corporation, Armonk, NY, USA) and cluster membership to determine the number of clusters for each choroidal parameter. Separability of the clusters was then tested using the Wilcoxson-sign ranked *t*-test. The clusters were then presented spatially in 8 × 8 topographical maps by colour-coding each grid location based on its respective cluster (Fig. 1E) and numerically as the median ± 95% CI value for all grid locations within the cluster.

Next, 8 × 8 grid data was analysed based on the 4 retinal quadrants. Grid locations were separated into the superior, inferior, nasal, and temporal quadrants and averaged (Fig. 1F). For grid locations that fell across two quadrants, the contribution of the grid to the average of the quadrant was scaled by 50%.

Finally, 8 × 8 grid data was analysed based on the ETDRS template. Grid locations were separated into the centre (1 mm), inner ring (3 mm) and outer ring (6 mm). Grid locations outside the ETDRS sectors were assigned to the extramacular region. As most grid locations fell across multiple ETDRS sectors, they were scaled based on the percentage of pixels of a grid location within an ETDRS sector. Analysis was repeated for individual inner ring/outer ring superior, inferior, temporal, and nasal subfields (Fig. 1G).

### Statistical analysis

Statistical analysis was performed using SPSS version 28.0 (IBM Corporation, Armonk, NY, USA) and GraphPad Prism (v8.0.2, GraphPad Software, San Diego, CA, USA). A p value of < 0.05 was considered significant. All data, including age, was treated as continuous unless specified otherwise (e.g., sex). All the data were subjected to normality test, failing which non-parametric tests were chosen and the values were expressed in median + 95% CI. Comparison of continuous values between clusters were performed using Wilcoxon’s Sign ranked *t*-test, while comparisons between ETDRS sectors and quadrants were performed using Friedman test with Dunn’s correction for multiple comparisons. Multivariable regressions were performed using Stepwise elimination method until significance was reached (*p* < 0.05). Specifically, age regressions were fitted into both quadratic and linear functions and the best fit model was selected using Run’s test.

## Results

### Study population

Three hundred and forty two participants were screened of which three hundred and nine eyes of 309 participants were included in this study. The demographic details of the population are provided in Table 1. Briefly, all participants were free of systemic vascular-related disorders (e.g. Diabetes, Hypertension, Cardiovascular disorders) and posterior segment diseases. Refractive error (RE) was ± 3D in < 40 years (91%) and ± 6D for > 40 years (9%) to account for variation secondary to age related changes. The overall ethnic distribution of the study population were 55% white population, 34% Asians and 7% other ethnic groups (Table 1).

**Table 1:** Demographics, ocular characteristics, and global mean values of CVI and area parameters of the study population.

Cohort	*n*	Age (median; IQR)	Sex (M: F; %)	Ethnicity (W: A:O: U; %)*		Image quality (median dB)		IOP (median mmHg)		Spherical Equivalent (median Dioptres; IQR)	CVI (%)	TCA (mm^2^)		LA (mm^2^)	SA (mm^2^)				
All Participants	309	48.4 (25)	60:40	55:34:7:4		30.6		15		0.00 (1.38)	66.4	0.21		0.14	0.071				
20–29 years old	47	24.2 (5)	57:43	43:49:4:6		32.0		14.6, 11		0.00 (1.88)	66.3	0.24		0.16	0.080				
30–39 years old	50	35.3 (5)	40:60	28:52:20:0		32.2		15.0, 6		-0.50 (2.13)	65.8	0.24		0.16	0.081				
40–49 years old	72	45.7 (4)	30:70	57:42:0:1		30.6		15.5, 3		-0.13 (1.22)	66.2	0.22		0.14	0.074				
50–59 years old	54	53.9 (6)	43:57	67:19:5:6		31.0		14.9, 4		0.00 (1.75)	67.4	0.21		0.14	0.067				
60–69 years old	50	64.0 (5)	46:54	60:24:6:10		29.0		15.1, 1		0.00 (2.59)	66.6	0.18		0.12	0.058				
> 70 years old	36	74.2 (5)	53:47	80:11:6:3		28.1		15.0, 1		0.03 (1.63)	66.3	0.17		0.11	0.057				

*W: white, A: Asian, O: Others U: Unknown.

### Primary analysis: spatial topography of choroidal vascularity index

- *Global analysis*

Individual grid values of CVI and area parameters (total choroidal area, TCA; luminal area, LA; stromal area; SA) were pooled and averaged to determine a global value across the whole macula region. Global mean CVI for all eyes was 66.4% [66.15, 66.70] and ranged between 65.8 − 67.4% when assessed within individual decade age groups with no significant difference between age groups (*p* = 0.82; Table 1). Mean TCA, LA and SA was 0.21mm^2^ [0.20, 0.215], 0.14 mm2 [0.136, 0.143] and 0.071mm^2^ [0.068, 0.072] respectively with decreasing values across each decade.

b) *Cluster analysis*

**Figure 2:**
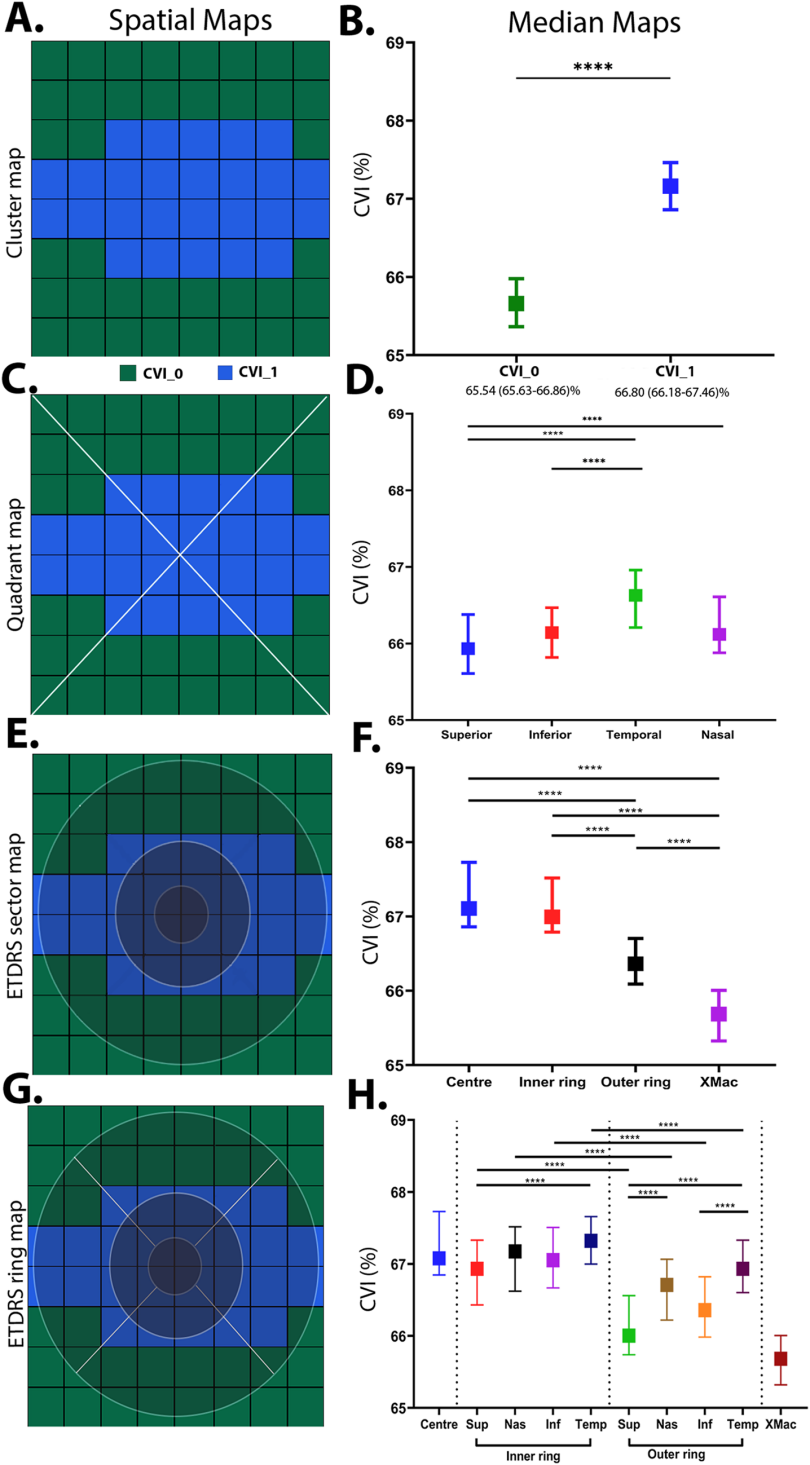
Spatial cluster topography of Choroidal vascularity index (CVI %) in different spatial topographies (**A**) cluster map, (**B**) cluster median distribution, (**C**) quadrant map, (**D**) quadrant median distribution, (**E**) ETDRS pooled ring map, (**F**) ETDRS median distribution, (**G**) ETDRS sectoral map, (**H**) ETDRS sectoral median distribution. All values are medians and error bars are 95% CI. Significance level *p < 0.05, **p < 0.01, *** *p* < 0.001, ****p < 0.0001.

Grid-wise values of CVI were grouped into statistically similar clusters, then presented as topographical maps (Fig. 2A) and graphs of cluster medians across all age groups (Fig. 2B). CVI displayed a concentric pattern with a central cluster of higher median value (66.8% [66.18, 67.46]) and an extramacular cluster of lower median value (65.63% [65.63, 66.86]).

c) *Quadrant analysis*

CVI was then assessed using standard clinical templates such as quadrant analysis to account for clusters extending in extramacular region in nasal temporal quadrants. Median CVI was significantly lower in the superior and inferior quadrants compared to the temporal quadrant (sup. vs. temp. difference: -0.19%, *p* = 0.0001; inf. vs. temp. difference: -0.46%, *p* = 0.0006) and in the superior quadrant compared to the nasal quadrant (difference: -0.7%, *p* = 0.0004). There was no significant difference in median CVI of the superior versus inferior quadrants (*p* = 0.47), inferior versus nasal (*p* = 0.15) or nasal versus temporal quadrants (*p* = 0.57, Fig. 2C-D).

d) *ETDRS template analysis*

Spatial differences were also analysed within the ring and sectors of the ETDRS template (Fig. 2E-H). CVI was greatest at the central sector (67.08% [66.85,67.73]) and inner ring (67.0% [66.79,67.52]) with no statistically significant difference between these regions (*p* = 0.26). This was followed by a significant decline in the outer ring (66.36% [66.09, 66.70]; *p* < 0.0001 for all comparisons) and extra macular region outside the ETDRS sectors (*p* < 0.0001; Supplementary Table S1; Fig. 2F).

As expected, CVI of all ETDRS sectors significantly decreased between their inner and outer ring counterpart (*p* < 0.001 for all comparisons, Fig. 2H). Differences in CVI were also observed between the superior versus temporal subfield of the inner ring (*p* < 0.01) and the superior versus nasal, superior versus temporal (*p* < 0.0001) and inferior versus temporal subfields in the outer ring (*p* < 0.0001).

### Secondary analysis: spatial topography of choroidal area parameters

- *Cluster analysis*

**Figure 3:**
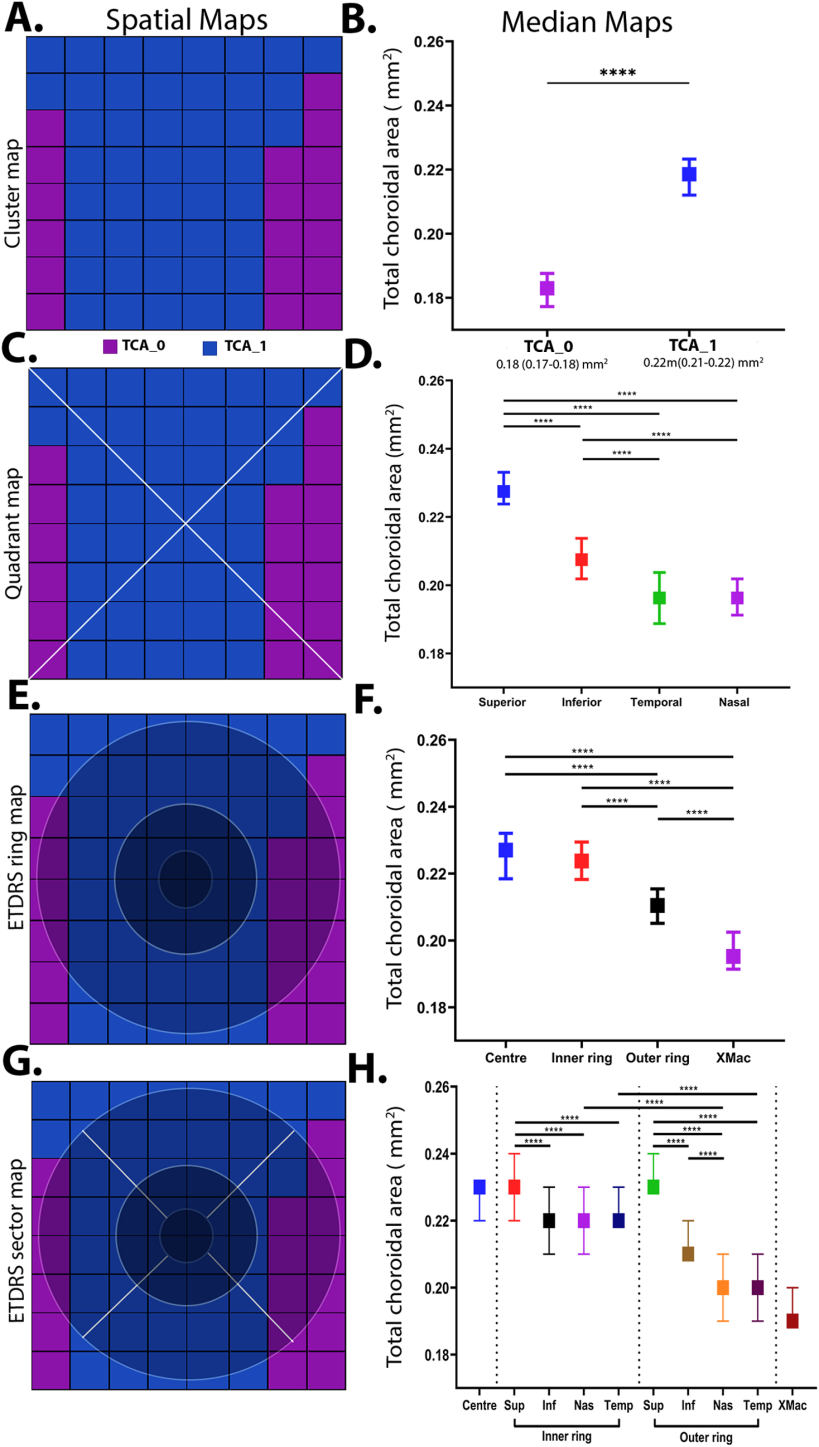
Spatial cluster topography of Total choroidal area (TCAmm^2^) in different spatial topographies, (**A**) cluster map, (**B**) cluster median distribution, (**C**) quadrant map, (**D**) quadrant median distribution, (**E**) ETDRS pooled ring map, (**F**) ETDRS median distribution, (**G**) ETDRS sectoral map, (**H**) ETDRS sectoral median distribution. All values are medians and error bars are 95% CI. Significance level *p < 0.05, **p < 0.01, *** *p* < 0.001, ****p < 0.0001.

**Figure 4:**
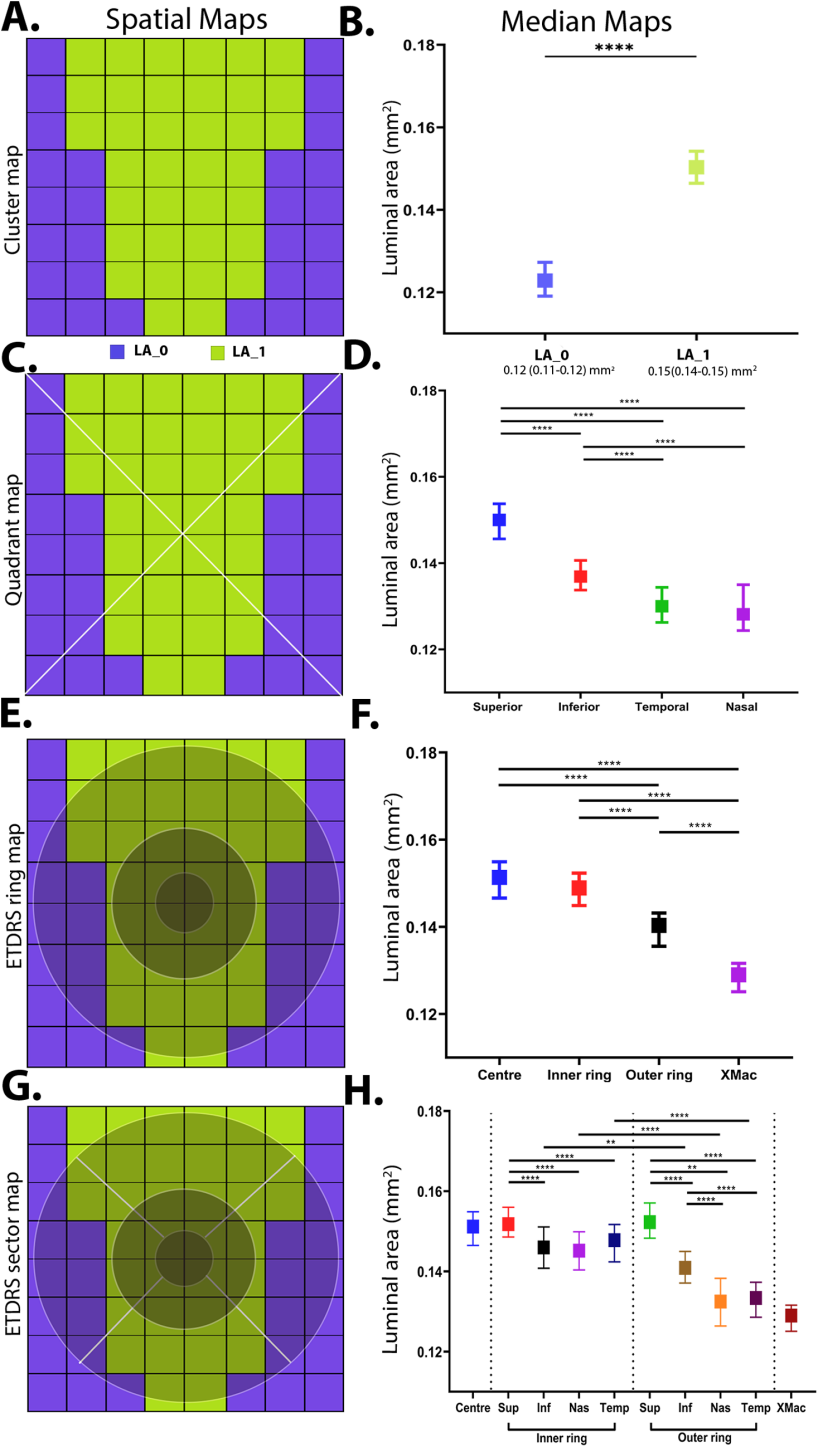
Spatial cluster topography of Luminal area (LA mm^2^) in different spatial topographies (**A**) cluster map, (**B**) cluster median distribution, (**C**) quadrant map, (**D**) quadrant median distribution, (**E**) ETDRS pooled ring map, (**F**) ETDRS median distribution, (**G**) ETDRS sectoral map **H**) ETDRS sectoral median distribution. All values are medians and error bars are 95% CI. Significance level: *p < 0.05, **p < 0.01, *** *p* < 0.001, ****p < 0.0001.

**Figure 5:**
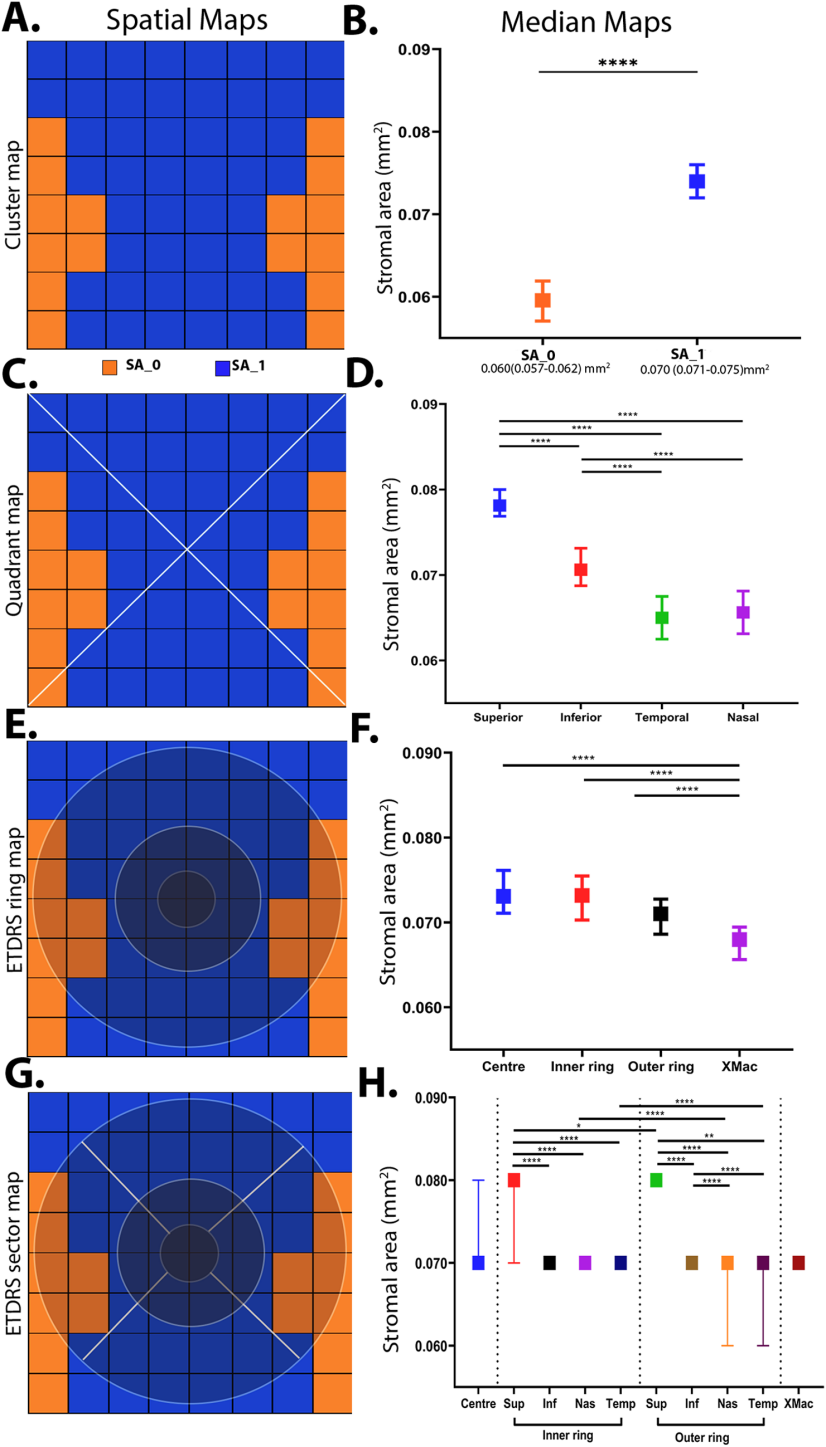
Spatial cluster topography of Stromal area (SA mm^2^) in different spatial topographies (**A**) cluster map, (**B**) cluster median distribution, (**C**) quadrant map, (**D**) quadrant median distribution, (**E**) ETDRS pooled ring map, (**F**) ETDRS median distribution, (**G**) ETDRS sectoral map, (**H**) ETDRS sectoral median distribution. All values are medians and error bars are 95% CI. Narrow range of median resulted in clipped error bars. Significance level: *p < 0.05, **p < 0.01, *** *p* < 0.001, ****p < 0.0001.

TCA displayed a single, central cluster that extended to the superior and inferior borders of the macula grid and a peripheral cluster mostly containing the extra-macular grid locations at the nasal and temporal retina (Fig. 3A). This central cluster displayed a significantly higher median value versus the extramacular cluster (TCA_central_: 0.22 [0.21–0.22] mm^2^ vs. TCA_xmac_: 0.18 [0.17–0.18] mm^2^, *p* < 0.001; Fig. 3B). LA and SA displayed a similar topography with higher central cluster with a superior-inferior bias and lessor extra-macular cluster (Figs. 4A-B and 5 A-B).

b) *Quadrant analysis*

Corresponding to the cluster map, the highest median values for TCA were observed in the superior quadrant, followed by the inferior, nasal and temporal quadrant (Fig. 3C-D). All quadrant medians for were significant differently from each other (all *p* < 0.0001) except the nasal and temporal quadrants. LA and SA displayed the same spatial pattern across retinal quadrants (Figs. 4 C-D and 5 C-D).

c) *ETDRS template analysis*

The ETDRS template revealed that the greatest TCA value was localised to the central (0.227 [0.220, 0.230] mm^2^) and inner ring (0.223 [0.218, 0.229] mm^2^) followed by a decline in the outer ring (0.210 [0.205, 0.215] mm^2^; *p* < 0.0001; Fig. 3E-F). Pooled values for the ETDRS inner rings were also significantly higher compared to the extra macular region outside the ETDRS sectors (*p* < 0.0001 for all comparisons; Supplementary Table S1). LA showed similar spatial findings with greater area in the central and inner rings (LA_central_: 0.151 [0.146, 0.154] mm^2^; LA_inner_: 0.149 [0.144, 0.152] mm^2^; *p* = 0.08) versus outer and extra macular region (*p* < 0.0001 for all comparisons; Fig. 4E-F). SA showed no significant differences between any pooled ETDRS rings (*p* ≥ 0.1 for all comparisons) however the central, inner, and outer rings were significantly different to the extra-macular region outside the ETDRS sectors (*p* < 0.001 for all comparisons; Supplementary Table S1, Fig. 5E-F).

For sectoral considerations, the superior subfield was significantly different to all other quadrants in both the inner (*p* < 0.00001–0.0005) and outer ring (*p* < 0.0001) for TCA (Supplementary Table S1; Fig. 3G-H). Between the inner and outer ring, the nasal and temporal subfields also showed significant decline in TCA (*p* < 0.0001 for all comparisons). LA and SA showed similar patterns in the inner ring with only the superior subfield being significantly greater than all other inner subfields (*p* < 0.05 for all comparisons, Figs. 4G-H and 5G-H). LA and SA of the outer ring subfields however were all significantly different to each other except between nasal and temporal subfields (*p* > 0.99). Between the two rings, LA of the inferior, nasal and temporal subfield significantly decreased from inner to outer ring (*p* < 0.05 for all comparisons) while for SA of the superior, nasal and temporal subfields were reduced significantly decreased between inner and outer ring (*p* < 0.0001).

## Association with ocular factors and age

**Figure 6:**
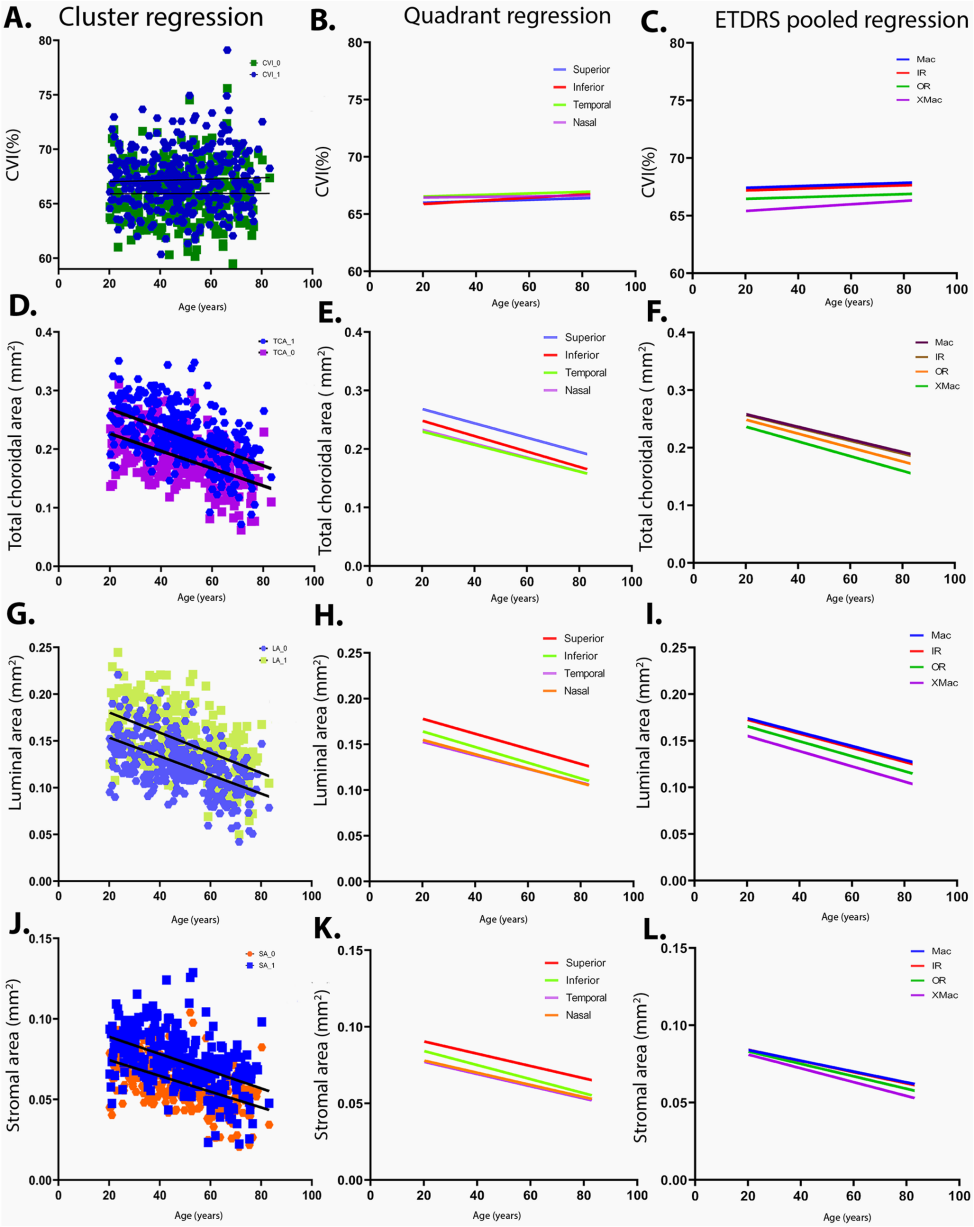
Regression equation of all Choroidal parameters across (**A**, **D**, **G**, **J**) Clusters (**B**, **E**, **H**, **K**) Quadrant regression (**C**, **F**, **I**, **L**) ETDRS pooled regression.

Multivariate regression indicated that CVI was not significantly associated with any variables including age across any spatial configuration (Fig. 6 A-C). Significant negative associations were observed between age and choroidal area parameters, TCA, LA, SA, for all spatial templates including clusters, quadrants and ETDRS sectors (Table 2). A significant positive association between refractive error and the temporal quadrant for LA, all ETDRS sectors for TCA and LA, and all clusters and quadrants for SA (*p* = 0.0001). A significant positive association between visual acuity and SA of the superior quadrant was also observed (*p* = 0.0001). No choroidal parameters showed any significant relationship with IOP, BCVA, ethnicity or sex.

**Table 2:** The multivariable regression of the cohort for different eccentricities for significant variables.

Eccentricities		TCA				LA				SA							
		Age(ß)	p value	RE(ß)	p value	Age(ß)	p value	RE(ß)	p value	Age(ß)	p value	RE(ß)	p value	Ethnicity (ß)	p value	Log Vision(ß)	p value
Cluster	Cluster_0	-0.558	< 0.01***	0.228	< 0.01***	-0.563	< 0.01**	0.561	< 0.01***	-0.514	< 0.01***	0.199	< 0.01***	0.154	**0.003**	-	-
	Cluster_1	-0.556	< 0.01***	0.233	< 0.01***	-0.224	< 0.01**	0.226	< 0.01***	-0.503	< 0.01***	0.226	< 0.01***	0.116	**0.026**	-	-
Quadrants	Superior quadrant	-0.9279	< 0.01***	-	-	-0.9238	< 0.01***	0.303	**0.0141**	-0.539	< 0.01***	0.19	< 0.01***	-		0.113	0.032**
	Inferior quadrant	-0.9279	< 0.01***	-	-	-0.9238	< 0.01***	-		-0.513	< 0.01***	0.221	< 0.01***	-		-	-
	Temporal quadrant	-0.9279	< 0.01***	-	-	-0.9279	< 0.01***	0.308	0.04***	-0.47	< 0.01***	0.239	< 0.01***	-		-	-
	Nasal quadrant	-0.9279	< 0.01***	-	-	-0.9279	< 0.01***	-	**-**	-0.493	< 0.01***	0.139	0.011**	-		-	-
ETDRS	Mac	-0.479	< 0.01***	0.252	< 0.01***	-0.5	< 0.01**	0.236	< 0.01***	- 0.363	< 0.01***	0.131	0.021**	-		-	-
	IRS	-0.488	< 0.01***	0.222	< 0.01***	-0.506	< 0.01**	0.208	< 0.01***	-0.379	< 0.01***	-		-		-	-
	IRN	-0.475	< 0.01***	0.246	< 0.01***	-0.497	< 0.01**	0.239	< 0.01***	- 0.375	< 0.01***	-		-		-	-
	IRI	-0.478	< 0.01***	0.255	< 0.01***	-0.494	< 0.01**	0.241	< 0.01***	- 0.380	< 0.01***	-		-		-	-
	IRT	-0.477	< 0.01***	0.259	< 0.01***	-0.495	< 0.01**	0.241	< 0.01***	- 0.367	< 0.01***	-		-		-	-
	ORS	-0.551	< 0.01***	0.204	< 0.01***	-0.556	< 0.01**	0.195	< 0.01***	-0.459	< 0.01***	-		-		-	-
	ORN	-0.458	< 0.01***	0.178	< 0.01***	-0.466	< 0.01**	0.18	< 0.01***	-0.418	< 0.01***	-		-		-	-
	ORI	-0.52	< 0.01***	0.228	< 0.01***	-0.526	< 0.01**	0.195	< 0.01***	-0.443	< 0.01***	-		-		-	-
	ORT	-0.472	< 0.01***	0.261	< 0.01***	-0.483	< 0.01**	0.26	< 0.01***	-0.391	< 0.01***	-		-		-	-
	Extramacular	-0.585	< 0.01***	0.191	< 0.01***	-0.584	< 0.01**	0.204	< 0.01***	-0.536	< 0.01***	-		-		-	-
Pooled ETDRS	Inner Ring	-0.512	0.01***	0.249	0.01***	-0.514	< 0.01***	0.240	< 0.01***	-0.459	< 0.01***	0.242	< 0.01***				
	Outer Ring	-0.572	0.01***	0.234	0.01***	-0.565	< 0.01***	0.234	< 0.01***	-0.538	< 0.01***	0.218	< 0.01***				

Abbreviations: IRS- Inner ring superior, IRN- Inner ring Nasal, IRI- Inner ring inferior, IRT-Inner ring temporal, ORS-Outer ring superior, ORN-Outer ring nasal, ORI-Outer ring inferior, ORT-Outer ring Temporal. Significance levels: *p<0.05, **p<0.01, ***p<0.001 ,****p<0.0001. Bold signifies values of statistical significance.

Based on significant age-related associations observed from multivariate analysis, regression models were applied to all choroidal area parameters and these were found to decline linearly with age(Fig. 6D-L) except CVI which displayed stability with progressing age. Regression models in clusters exhibited slightly different rates of areal decline for TCA (0.00148–0.00161 mm^2^/year), LA (0.0010–0.00108 mm^2^/year) and SA (0.00049–0.00053 mm^2^/year, p = < 0.0001, Supplementary Table S2). The rate of change across quadrants was highest inferiorly for all choroidal parameters (TCA:-0.001318, LA:-0.000858, SA:-0.0004602 mm^2^/ annum) followed by the superior, nasal and temporal quadrants. The rate of change was significantly different between the quadrants (*p* < 0.0001).

The decline with age across ETDRS sectors in all parameters exhibited lesser magnitude of decline at the centre (TCA: -0.00143, LA:-0.00097, SA:-0.00045 mm^2^/annum, Supplementary Table S2), followed by inner ring, outer ring then extra macular region and greater magnitude of decline at the inferior quadrant in all rings (*P* < 0.0001) for all comparisons).

## Discussion

This study found topographical variations in CVI with greatest magnitude in the centre and lesser magnitude in extramacular region but displayed no quadrant bias. TCA, LA and SA, similarly, exhibited the greatest magnitude at the central macula and additionally, a bias towards the superior and inferior quadrants. Multiple linear regression and age-based regressions indicated that CVI was not associated with any ocular factors nor age-related decline. On the other hand, all area-based parameters exhibited declined with increasing age and at significantly greater rates within the inferior ETDRS sectors and quadrant. These findings suggest that accurate assessment of choroidal area requires spatial and temporal considerations, while CVI may be robust against the latter.

Spatial analysis of CVI indicated central vs. extramacular gradient suggesting a higher luminal versus stromal area in the central retina. While this was consistent with our previous work^7^, it contrasts Singh et al^27^. who found reduced CVI at the macula and Breher et al^28^. and Agrawal et al. 2017^29^ reported minimal variation in CVI at various eccentricities. These discrepancies may be due to methodological limitations in these studies, whereby partial CVI values were calculated from single, representative B-scans. This current study however used a spatial-refined analysis across a high density, OCT volume scan and therefore may have better detected subtle topographical variation of choroidal parameters across all eccentricities.

All spatial analyses of choroidal area parameters in this study also indicated greater choroidal area in the central macula. This is consistent with in vivo studies that indicate the macula is structurally rich in choroidal vasculature compared to the peripheral retina^30^, likely to accommodate the greater photoreceptor density^31^of the central retina and corresponding metabolic demand^32,33^. Choroidal area parameters also demonstrated a superior to inferior bias, a spatial deviation which has previously been observed for choroidal area^7^and normal retinal vasculature^33^and may relate to the bi-arch choroidal vascular pattern noted in histological studies and similar to retinal vasculature, with superior and inferior branches across the posterior poles^34^. This superior-inferior bias appears to have physiological implications with higher rates of age-related regression noted in the inferior quadrants and rings for all choroidal parameters similar to previous work^7^. This bias may also explain spatial differences in chorio-retinal pathologies such as greater upper versus lower vortex vein filling in central serous chorioretinopathy^35^.

We found that CVI was found to be independent of all physiological factors tested including age, IOP, sex, and refractive error while all the individual choroidal area parameters (LA, SA, and TCA) were negatively correlated with age. This suggests that the rate of change remains consistent for all area components across all macula locations giving stable CVI in a healthy population. The role of normal aging on CVI is a current topic of debate with a number of studies suggesting CVI declines with age^7,36,37,38,39,40,41^while others support the absence of a correlation, similar to this study^4,15,42,43,44,45,46,47,48,49^. A meta-analysis of studies reporting CVI for healthy populations also suggest no significant association between age and CVI^50^. Reasons for these discrepancies could again be methodological (i.e., scan selection, OCT device, grading protocol) or sample based, considering most studies assess CVI within small homogenous populations. The present study however assessed a large, multi-ethnic, multi-decade, normative population with strict inclusion criteria including no systemic vascular related diseases, no smoking history, no ocular diseases and limited refractive error range. Hence, the null correlation between age and CVI in this study is likely to be robust and reiterates the value of CVI as a metric for assessment of the choroidal angioarchitecture based on its rigidity against physiological factors.

All choroidal area parameters declined linearly with age which aligned with in vivo and histological studies which describe decreased choroidal blood flow, choriocapillaris thickness, and overall choroidal thickness from the 3rd – 4th decade of life^51,52^. We also observed significant correlations between TCA and LA and refractive error when analysed according to clusters and ETDRS-based spatial analyses, but not quadrant analyses. Several studies have established reduction in choroidal elements in myopia^14,53^. Wallman^54^and Swiatczak et al^55^. have also demonstrated the ability of the macular choroid to locally vary its thickness (hence changes in TCA) according to myopic or hyperopic stimuli, which may explain why changes were accentuated in clusters and ETDRS sectors (with higher spatial density) but not quadrants.

There were a few limitations in this study. We quantified choroidal parameters from SD-OCT images which may have limited visualization of the choroid and its scleral boundary due to limited signal penetration. Future work using other OCT imaging modes such as swept-source OCT may help confirm the impact of this. Secondly, due to the retrospective nature of the study, the time of OCT measurement could not be standardized; therefore, the effect of diurnal variation could not be ascertained. Some physiological factors such as systemic vascular-related diseases were also ascertained based on self-reporting and therefore may not have been as reliable as objective measurement. We attempted to mitigate the impact of these limitations by employing strict selection criteria however future work could include further characterisation of individuals to confirm normal health of the population. Similarly, axial length measurements were unavailable to correct for individual ocular magnification, however our primary outcome measure, CVI being a ratio, is less likely to be affected by this. To reduce the effect on area parameters, refractive error criteria was fixed to^56^:91% of our participants had mild refractive error range and 9% of our participants had moderate myopic changes secondary to aging changes.

## Conclusions

This study found that the choroidal angioarchitecture exhibits topographical variations, with greater TCA, LA, and SA areas towards the central macula and significant differences between superior and inferior macula. Individual choroidal area parameters were associated with myopia in certain segments and significantly declined with age across all defined spatial analyses. In a homogenous healthy population, these changes are proportional in all choroidal parameters, resulting in stable CVI from the 2nd to the 7th decade of life. CVI was not associated with refractive error, IOP, ethnicity or visual acuity, highlighting its utility a valuable tool. CVI is also independent of ocular factor but changes in different eccentricities. Hence future studies involving choroidal measurement should be mindful of spatial topographical changes.

## Electronic supplementary material

Below is the link to the electronic supplementary material.

Supplementary Material 1

## Data Availability

The dataset generated during and/or analysed during the current study are available from the corresponding author on reasonable request.

## References

[1] Branchini, L. A. et al. Analysis of choroidal morphologic features and vasculature in healthy eyes using spectral-domain optical coherence tomography. *Ophthalmology*. **120**, 1901–1908 (2013). 23664466 10.1016/j.ophtha.2013.01.066 PMC3744589

[2] Sonoda, S. et al. Choroidal structure in normal eyes and after photodynamic therapy determined by binarization of optical coherence tomographic images. *Invest. Opthalmology Visual Sci. ***55**, 3893 (2014). 10.1167/iovs.14-14447 24894395

[3] Agrawal, R. et al. Choroidal vascularity index (CVI) - a novel optical coherence tomography parameter for monitoring patients with Panuveitis? *PLoS ONE.* **11**, e0146344 (2016). 10.1371/journal.pone.0146344 PMC4713828 26751702

[4] Agrawal, R. et al. Exploring choroidal angioarchitecture in health and disease using choroidal vascularity index. *Prog. Retin. Eye Res. ***77**, 100829 (2020). 31927136 10.1016/j.preteyeres.2020.100829

[5] Koh, L. H., Agrawal, R., Khandelwal, N., Sai Charan, L. & Chhablani, J. Choroidal vascular changes in age-related Macular Degeneration. *Acta Ophthalmol. ***95**, e597–601 (2017). 28391615 10.1111/aos.13399

[6] Kongwattananon, W., Kumar, A., Oyeniran, E., Sen, H. N. & Kodati, S. Changes in Choroidal Vascularity index (CVI) in intermediate uveitis. *Translational Vis. Sci. Technol. ***10**, 33 (2021). 10.1167/tvst.10.14.33 PMC8727317 34967835

[7] Nivison-Smith, L. et al. Normal aging changes in the choroidal angioarchitecture of the Macula. *Sci. Rep. ***10**, 10810 (2020). 10.1038/s41598-020-67829-2 PMC7331638 32616774

[8] Agarwal, A. et al. Choroidal structural changes in tubercular multifocal Serpiginoid Choroiditis. *Ocul. Immunol. Inflamm. ***26**, 838–844 (2018). 29020533 10.1080/09273948.2017.1370650

[9] Chun, H. et al. Choroidal vascularity index change in macular telangiectasia type 2. *PLoS ONE. ***17**, e0262112 (2022). 10.1371/journal.pone.0262112 PMC8989206 35389993

[10] Tan, K. et al. Choroidal vascularity index – a novel optical coherence tomography parameter for disease monitoring in diabetes mellitus? *Acta Ophthalmol. ***94**,e612-e616 (2016). 10.1111/aos.13044 27151819

[11] Wang, Y. M. et al. Characterization of macular choroid in normal-tension glaucoma: a swept‐source optical coherence tomography study. *Acta Ophthalmol. ***99**, e1421–e1429 (2021). 33675169 10.1111/aos.14829

[12] Yang, J. et al. CVIS: automated OCT-scan-based software application for the measurements of choroidal vascularity index and choroidal thickness. *Acta Ophthalmol. ***100**, e1553–e1560 (2022). 35415874 10.1111/aos.15152

[13] Azuma, K. et al. Assessment of the choroidal structure in pregnant women in the first trimester. *Sci. Rep. ***11**, 4629 (2021). 33633327 10.1038/s41598-021-84204-x PMC7907119

[14] Alis, G. M. & Alis, A. Choroidal vascularity index in adults with different refractive status. *Photodiagn. Photodyn. Ther. ***36**, 102533 (2021). 10.1016/j.pdpdt.2021.102533 34520880

[15] Parisi, V. et al. Macular functional and morphological changes in intermediate age-related maculopathy. *Invest. Opthalmol. Vis. Sci. ***61**, 11 (2020). 10.1167/iovs.61.5.11 PMC7405611 32396630

[16] Yip, V. C. H. et al. A longitudinal study of choroidal changes following cataract surgery in patients with diabetes. *Diabetes Vasc. Dis. Res. ***16**, 369–377 (2019). 10.1177/1479164119841536 31007056

[17] Wang, Y. et al. Vascular changes of the choroid and their correlations with visual acuity in pathological myopia. *Investig. Ophthalmol. Vis. Sci. ***63**, 20–20 (2022). 10.1167/iovs.63.12.20 PMC9672896 36378132

[18] Goud, A. et al. New insights on choroidal vascularity: a comprehensive topographic approach. *Invest. Opthalmol. Vis. Sci. ***60**, 3563 (2019). 10.1167/iovs.18-26381 31419299

[19] Buzzelli, M. Modifiable areal unit problem. *Int. Encyclopedia Hum. Geogr.* 169–173. 10.1016/b978-0-08-102295-5.10406-8 (2020).

[20] Tong, J. et al. Development of a spatial model of age-related change in the macular ganglion cell layer to predict function from structural changes. *Am. J. Ophthalmol. ***208**, 166–177 (2019). 31078539 10.1016/j.ajo.2019.04.020 PMC6842123

[21] Yoshioka, N. et al. Pattern recognition analysis of age-related retinal ganglion cell signatures in the human eye. *Invest. Opthalmol. Vis. Sci. ***58**, 3086–3099 (2017). 10.1167/iovs.17-21450 PMC5482244 28632847

[22] Trinh, M. et al. Macula ganglion cell thickness changes display location-specific variation patterns in intermediate age-related macular degeneration. *Invest. Opthalmol. Vis. Sci. ***61**, 2 (2020). 10.1167/iovs.61.3.2 PMC7401429 32150251

[23] Trinh, M., Khou, V., Zangerl, B., Kalloniatis, M. & Nivison-Smith, L. Modelling normal age-related changes in individual retinal layers using location-specific OCT analysis. *Sci. Rep. ***11**, 558 (2021). 10.1038/s41598-020-79424-6 PMC7804110 33436715

[24] Choi, A. Y. et al. Contrast sensitivity isocontours of the central visual field. *Sci. Rep. ***9**, 11603 (2019). 10.1038/s41598-019-48026-2 PMC6691009 31406197

[25] Wang, H. & Kalloniatis, M. Clinical outcomes of the centre for eye health: an intra-professional optometry-led collaborative eye care clinic in Australia. *Clin. Exp. Optom. ***104**, 795–804 (2021). 33689627 10.1080/08164622.2021.1878821

[26] Salmon, A. E., Sajdak, B. S., Atry, F. & Carroll, J. Axial scaling is independent of ocular magnification in OCT images. *Invest. Opthalmol. Vis. Sci. ***59**, 3037–3040 (2018). 10.1167/iovs.17-23549 PMC6005622 30025118

[27] Singh, S. R. et al. Wide-field choroidal vascularity in healthy eyes. *Am. J. Ophthalmol. ***193**, 100–105 (2018). 29958821 10.1016/j.ajo.2018.06.016

[28] Breher, K., Terry, L., Bower, T. & Wahl, S. Choroidal biomarkers: a repeatability and topographical comparison of choroidal thickness and choroidal vascularity index in healthy eyes. *Transl. Vis. Sci. Technol. ***9**, 8 (2020). 33133771 10.1167/tvst.9.11.8 PMC7552934

[29] Agrawal, R. et al. Influence of scanning area on choroidal vascularity index measurement using optical coherence tomography. *Acta Ophthalmol. ***95**, e770-e775 (2017). 10.1111/aos.13442 28470942

[30] Hayreh, S. S. In vivo choroidal circulation and its watershed zones.* Eye*. **4**, 273–289 (1990) 2199236 10.1038/eye.1990.39

[31] Curcio, C. A., Sloan, K. R., Kalina, R. E. & Hendrickson, A. E. Human photoreceptor topography. *J. Comp. Neurol. ***292**, 497–523 (1990). 2324310 10.1002/cne.902920402

[32] Shahidi, A. M., Patel, S. R., Flanagan, J. G. & Hudson, C. Regional variation in human retinal vessel oxygen saturation. *Exp. Eye Res. ***113**, 143–147 (2013). 23791637 10.1016/j.exer.2013.06.001

[33] Tomita, R. et al. Differences in blood flow between superior and inferior retinal hemispheres. *Invest. Opthalmology Visual Sci. ***61**, 27 (2020). 10.1167/iovs.61.5.27 PMC7405729 32421146

[34] Hayreh, S. S. Posterior ciliary artery circulation in health and disease the Weisenfeld Lecture. Investigative Opthalmology & Visual Science. **45**, 749–757 (2004) 10.1167/iovs.03-0469 14985286

[35] Kishi, S. et al. Geographic filling delay of the choriocapillaris in the region of dilated asymmetric vortex veins in central serous chorioretinopathy. *PLoS ONE.* **13**, e0206646 (2018). 10.1371/journal.pone.0206646 PMC6226146 30412594

[36] Koçak, N., Subaşı, M. & Yeter, V. Effects of age and binarising area on choroidal vascularity index in healthy eyes: an optical coherence tomography study. *Int. Ophthalmol. ***41**, 825–834 (2020). 33170421 10.1007/s10792-020-01636-6

[37] Wei, X. et al. Comparison of choroidal vascularity markers on optical coherence tomography using two-image binarization techniques. *Invest. Opthalmol. Vis. Sci. ***59**, 1206–1211 (2018). 10.1167/iovs.17-22720 29625441

[38] Wei, X. et al. Choroidal structural analysis and Vascularity Index in retinal dystrophies. *Acta Ophthalmol. ***97**, e116–e121 (2019). 30178525 10.1111/aos.13836

[39] Verma, A. et al. Age-related alterations of the Macular Choroid in healthy eyes assessed by swept-source Optical Coherence Tomography Angiography. *Ophthalmic Surg. Lasers Imaging Retina*. **54**, 526–534 (2023). 37642439 10.3928/23258160-20230728-01

[40] Ma, F., Bai, Y., Duan, J., Liang, Y. & Shang, Q. Validation of reliability, repeatability and consistency of three-dimensional choroidal vascular index. *Sci. Rep. ***14**, 1576 (2024). 38238371 10.1038/s41598-024-51922-x PMC10796765

[41] Xuan, M. et al. Distribution and determinants of choroidal vascularity index in healthy eyes from deep-learning choroidal analysis: a population-based SS-OCT study. *Br. J. Ophthalmol. ***108**, 546–551 (2024). 37001972 10.1136/bjo-2023-323224

[42] Agrawal, R. et al. Choroidal vascularity index as a measure of vascular status of the choroid: measurements in healthy eyes from a population-based study. *Sci. Rep. ***6**, 21090 (2016). 26868048 10.1038/srep21090 PMC4751574

[43] Cheong, K. X. et al. Three-dimensional modelling of the choroidal angioarchitecture in a multi-ethnic Asian population. *Sci. Rep. ***12**, 3831 (2022). 35264578 10.1038/s41598-022-07510-y PMC8907174

[44] Zhou, H. et al. Age-related changes in choroidal thickness and the volume of vessels and stroma using swept-source OCT and fully automated algorithms. *Ophthalmol. Retina. ***4**, 204–215 (2020). 32033714 10.1016/j.oret.2019.09.012 PMC7812781

[45] Giannaccare, G. et al. Choroidal vascularity index quantification in geographic atrophy using binarization of enhanced-depth imaging optical coherence tomographic scans. *Retina. ***40**, 960–965 (2020). 30676528 10.1097/IAE.0000000000002459

[46] Iovino, C. et al. Effects of different mydriatics on the choroidal vascularity in healthy subjects. *Eye. ***35**, 913–918 (2020). 32467635 10.1038/s41433-020-0995-9 PMC8026984

[47] Magesan, K., Sachidanandam, R., Verma, A. & Biswas, J. Retino-choroidal evaluation of the macular region in eyes with tubercular serpiginous-like choroiditis using swept-source optical coherence tomography angiography. *Int. Ophthalmol. ***42**, 2651–2664 (2022). 35364747 10.1007/s10792-022-02254-0

[48] Sidorczuk, P., Obuchowska, I., Konopinska, J. & Dmuchowska, D. A. Correlation between choroidal vascularity index and outer retina in patients with diabetic retinopathy. *J. Clin. Med. ***11**, 3882 (2022). 35807164 10.3390/jcm11133882 PMC9267134

[49] Tan, R. et al. Choroidal vascularity index in Retinitis Pigmentosa: an oct study. Ophthalmic surgery. *Lasers Imaging Retina. ***49**, 191–197 (2018). 10.3928/23258160-20180221-07 29554387

[50] Kumar, M. et al. Choroidal vascularity index is independent of ocular and image-based influencers in healthy population: a systematic review and meta analysis. Manuscript in preparation. (2024). 10.1038/s41598-025-10384-5 PMC12311116 40739311

[51] Chirco, K. R., Sohn, E. H., Stone, E. M., Tucker, B. A. & Mullins, R. F. Structural and molecular changes in the aging choroid: implications for age-related macular degeneration. *Eye. ***31**, 10–25 (2016). 27716746 10.1038/eye.2016.216 PMC5233940

[52] Fragiotta, S. et al. Choroidal vasculature changes in age-related macular degeneration: from a molecular to a clinical perspective. *Int. J. Mol. Sci. ***23**, 12010 (2022). 36233311 10.3390/ijms231912010 PMC9570412

[53] Liu, M. et al. Differences in choroidal responses to near work between myopic children and young adults. *Eye Vis. ***11**, 12 (2024). 10.1186/s40662-024-00382-5 PMC10986059 38561862

[54] Wallman, J. et al. Moving the retina: choroidal modulation of refractive state. *Vision. Res. ***35**, 37–50 (1995). 7839608 10.1016/0042-6989(94)e0049-q

[55] Swiatczak, B., Schaeffel, F. & Calzetti, G. Imposed positive defocus changes choroidal blood flow in young human subjects. *Graefe’s Archive Clin. Exp. Ophthalmol. ***261**, 115–125 (2022). 10.1007/s00417-022-05842-z PMC9803748 36171460

[56] Gaurisankar, Z. S. et al. Correlations between ocular biometrics and refractive error: a systematic review and meta-analysis. *Acta Ophthalmol. ***97**, 735 – 43 (2019). 10.1111/aos.14208 31386806

