# Supplementary material for: Age related grid-wise spatial analysis of choroidal parameters in well characterised healthy population
Source: Sci Rep. 2024 Nov 4;14:26592. doi: 10.1038/s41598-024-76844-6 (PMC11535518; doi:10.1038/s41598-024-76844-6)
Supplement: Supplementary file 1 — Supplementary Material 1 [file 41598_2024_76844_MOESM1_ESM.docx]

Supplementary Table S1. Choroidal parameters across ETDRS segments.

p-values calculated by Friedman with Dunn’s correction for multiple comparison.

| **Choroidal Parameter** | **Segment** | | **Median** | **95% CI** | **P value** | | | | | | | | | | |
| --- | --- | --- | --- | --- | --- | --- | --- | --- | --- | --- | --- | --- | --- | --- | --- |
|  |  |  |  |  | **Pooled ETDRS** | | | **Inner ring** | | | | **Outer ring** | | | |
|  |  |  |  |  | **c.f. centre** | **c.f. inner ring** | **c.f. outer ring** | **c.f. superior** | **c.f. nasal** | **c.f.**  **inferior** | **c.f. Temporal** | **c.f. superior** | **c.f. nasal** | **c.f.**  **inferior** | **c.f. Temporal** |
| CVI(%) | Centre |  | 67.08 | 66.85-67.73 | - |  |  | **0.09****** | >0.99 | >0.99 | >0.99 | **<0.01***** | **<0.01***** | **<0.01***** | **0.02**^****^ |
|  | Inner ring | Whole | 67.00 | 66.79-67.52 | 0.26 | - |  | - | - | - | - | - | - | - | - |
|  |  | Superior | 66.93 | 66.43-67.33 | - | - | - | - | 0.12 | 0.79 | **0.0013***** | **<0.01***** | >0.99 | **<0.01***** | >0.99 |
|  |  | Nasal | 67.17 | 66.62-67.52 | - | - | - | - | - | >0.99 | >0.99 | **<0.01***** | **<0.01***** | **<0.01***** | **0.036*** |
|  |  | Inferior | 67.05 | 66.67-67.51 | - | - | - | - | - | - | >0.99 | **<0.01***** | **<0.01***** | **<0.01***** | 0.31 |
|  |  | Temporal | 67.32 | 67.0-67.66 | - | - | - |  |  |  |  | **<0.01***** | **<0.01***** | **<0.01***** | **0.03***** |
|  | Outer Ring | Whole | 66.36 | 66.09-66.70 | **<0.01***** | **<0.01***** | **-** | - | - | - | - | - | - | - | - |
|  |  | Superior | 66.00 | 65.74-66.56 | - | - | - | - | - | - | - | - | **<0.01***** | >0.99 | **<0.01***** |
|  |  | Nasal | 66.71 | 66.22-67.07 | - | - | - | - | - | - | - | - | - | 0.399 | >0.99 |
|  |  | Inferior | 66.36 | 65.98-66.82 | - | - | - | - | - | - | - | - | - | - | **0.02***** |
|  |  | Temporal | 66.93 | 66.60-67.33 | - | - | - | - | - | - | - | - | - | - | - |
|  | Extramacula |  | 65.68 | 65.32-66.01 | **<0.01***** | **<0.01***** | **<0.01***** | **<0.01***** | **<0.01***** | **<0.01***** | **<0.01***** | **0.0478** | **<0.01***** | **<0.01***** | **<0.01***** |
| TCA**(**mm2**)** | Centre |  | 0.227 | 0.220-0.230 | - | - |  | 0.22 | >0.99 | >0.99 | 0.52 | **0.020**** | **<0.01***** | 0.26 | **<0.01***** |
|  | Inner ring | Whole | 0.223 | 0.218-0.229 | 0.59 | - |  | - | - | - | - | - | - | - | - |
|  |  | Superior | 0.228 | 0.220-0.240 | - | - | - | - | **<0.01***** | **0.05***** | **<0.01***** | >0.99 | **<0.01***** | **<0.01***** | **<0.01***** |
|  |  | Nasal | 0.216 | 0.210-0.230 | - | - | - | - | - | >0.99 | >0.99 | **<0.01***** | **<0.01***** | >0.99 | **<0.01***** |
|  |  | Inferior | 0.219 | 0.210-0.230 | - | - | - | - | - | - | >0.99 | **<0.01***** | **<0.01***** | >0.99 | **<0.01***** |
|  |  | Temporal | 0.222 | 0.220-0.230 | - | - | - | - | - | - | - | **<0.01***** | **<0.01***** | >0.99 | **<0.01***** |
|  | Outer Ring | Whole | 0.210 | 0.205-0.215 | **<0.01***** | **<0.01***** | - | - | - | - | - | - | - | - | - |
|  |  | Superior | 0.232 | 0.230-0.240 | - | - | - | - | - | - | - | - | **<0.01***** | **<0.01***** | **<0.01***** |
|  |  | Nasal | 0.199 | 0.190-0.210 | - | - | - | - | - | - | - | - | **-** | **<0.01***** | >0.99 |
|  |  | Inferior | 0.212 | 0.210-0.220 | - | - | - | - | - | - | - | - | - | - | **<0.01***** |
|  |  | Temporal | 0.199 | 0.190-0.210 | - | - | - | - | - | - | - | - | - | - | - |
|  | Extramacula |  | 0.194 | 0.191-0.202 | **<0.01***** | **<0.01***** | **<0.01***** | **<0.01***** | **<0.01***** | **<0.01***** | **<0.01***** | **<0.01***** | >0.99 | **<0.01***** | >0.99 |
| LA(mm2**)** | Centre |  | 0.151 | 0.146-0.154 | - | - |  | 0.07 | **0.028*** | 0.26 | **0.036***** | **0.028** | **<0.01***** | **<0.01***** | **<0.01***** |
|  | Inner ring | Whole | 0.149 | 0.144-0.152 | 0.08 | - |  | - | - | - | - | - | - | - | - |
|  |  | Superior | 0.152 | 0.148-0.156 | - | - | - | - | **<0.01***** | **<0.01***** | **<0.01***** | >0.99 | **<0.01***** | **<0.01***** | **<0.01***** |
|  |  | Nasal | 0.145 | 0.140-0.149 | - | - | - | - | - | >0.99 | >0.99 | **<0.01***** | **<0.01***** | 0.16 | **<0.01***** |
|  |  | Inferior | 0.146 | 0.140-0.151 | - | - | - | - | - | - | >0.99 | **<0.01***** | **<0.01***** | **0.0156** | **<0.01***** |
|  |  | Temporal | 0.147 | 0.142-0.151 | - | - | - | - | - | - | - | **<0.01***** | **<0.01***** | 0.75 | **<0.01***** |
|  | Outer Ring | Whole | 0.140 | 0.135-0.143 | **<0.01***** | **<0.01***** | - | - | - | - | - | - | - | - | - |
|  |  | Superior | 0.152 | 0.148-0.157 | - | - | - | - | - | - | - | - | **<0.01***** | **<0.01***** | **<0.01***** |
|  |  | Nasal | 0.132 | 0.126-0.138 | - | - | - | - | - | - | - | - | - | **<0.01***** | >0.99 |
|  |  | Inferior | 0.140 | 0.137-0.145 | - | - | - | - | - | - | - | - | - | - | **<0.01***** |
|  |  | Temporal | 0.133 | 0.128-0.137 | - | - | - | - | - | - | - | - | - |  | - |
|  | Extramacula |  | 0.129 | 0.125-0.131 | **<0.01***** | **<0.01***** | **<0.01***** | **<0.01***** | **<0.01***** | **<0.01***** | **<0.01***** | **<0.01***** | 0.12 | **<0.01***** | 0.86 |
| SA**(**mm2) | Centre |  | 0.073 | 0.070-0.080 | - |  |  | **0.05***** | >0.99 | >0.99 | 0.56 | **<0.01***** | **<0.01***** | >0.99 | **<0.01***** |
|  | Inner ring | Whole | 0.073 | 0.070-0.075 | >0.99 | - |  | - | - | - | - | - | - | - | **<0.01***** |
|  |  | Superior | 0.080 | 0.070-0.080 | - | - | - | - | **<0.01***** | **<0.01***** | **<0.01***** | **0.017** | **<0.01***** | **<0.01***** | **<0.01***** |
|  |  | Nasal | 0.070 | 0.070-0.070 | - | - | - | - | - | >0.99 | >0.99 | **<0.01***** | **<0.01***** | >0.99 | **<0.01***** |
|  |  | Inferior | 0.070 | 0.070-0.070 | - | - | - | - | - | - | >0.99 | **<0.01***** | **<0.01***** | >0.99 | **<0.01***** |
|  |  | Temporal | 0.070 | 0.070-0.070 | - | - | - | - | - | - | - | **<0.01***** | **<0.01***** | >0.99 | **<0.01***** |
|  | Outer Ring | Whole | 0.070 | 0.068-0.072 | 0.14 | 0.13 | - | - | - | - | - | - | - | - | - |
|  |  | Superior | 0.080 | 0.060-0.070 | - | - | - | - | - | - | - | - | **<0.01***** | **<0.01***** | **<0.01***** |
|  |  | Nasal | 0.070 | 0.070-0.070 | - | - | - | - | - | - | - | - | **<0.01***** | **<0.01***** | >0.99 |
|  |  | Inferior | 0.070 | 0.060-0.070 | - | - | - | - | - | - | - | - | **<0.01***** | **<0.01***** | **<0.01***** |
|  |  | Temporal | 0.070 | 0.060-0.070 | - | - | - | - | - | - | - | - | - | - | - |
|  | Extramacula |  | 0.070 | 0.060-0.070 | **<0.01***** | **<0.01***** | **<0.01***** | **<0.01***** | **<0.01***** | **<0.01***** | **<0.01***** | **<0.01***** | >0.99 | **<0.01****** | 0.83 |

*p<0.05, **p<0.01, *** p<0.001 ,****p<0.0001, c.f.- cumulative frequency

Supplementary Table S2. Regression equations for various eccentricities

p-values calculated by Wilcoxon’s signed rank test for cluster and Friedman with Dunn’s correction for multiple comparison for quadrant and ETDRS template.

| **Eccentricities** | | **TCA** | | **LA** | | **SA** | | **p value** |
| --- | --- | --- | --- | --- | --- | --- | --- | --- |
|  |  |  |  |  |  |  |  |  |
|  |  | **Age correction**  **regression function** | **Rate of change**  **(mm^2^/year)** | **Age correction**  **regression function** | **Rate of change**  **(mm^2^/year)** | **Age correction**  **regression function** | **Rate of change**  **(mm^2^/year)** |  |
| **Clusters** | Cluster_0 | y= -0.001483x+0.2561 | -0.00148 | y=-0.0009976x+0.1735 | -0.00100 | y=-0.0004901x+0.08427 | -0.0049 | **<0.01****** |
|  | Cluster_1 | y= -0.001610x+0.3006 | -0.00161 | y=-0.001077x+0.2019 | -0.00108 | y=-0.0005340x+0.09961 | -0. 00053 |  |
| **Quadrants** | Superior  quadrant | y= -0.001225x-0.2930 | -0.001225 | y= -0.0008240x+0.1945 | -0.000824 | y=-0.0004017x+0.09852 | -0. 000402 | **<0.01****** |
|  | Inferior  quadrant | y= -0.001318x+0.2748 | -0.001318 | y=-0.0008580x+0.1814 | -0.000858 | y=-0.0004602x +0.0934 | -0. 00046 |  |
|  | Temporal  quadrant | Y= -0.001142x+0.2527 | -0.001142 | y=-0.0007423x+0.1676 | -0. 0007423 | y=-0.0003998x+0.08517 | -0. 00039 |  |
|  | Nasal  quadrant | y= -0.001188x+0.2567 | -0.001188 | y=-0.0007871x+0.1707 | -0. 000787 | y=-0.0003978x+0.08594 | -0. 00039 |  |
| **ETDRS** | Centre | y= -0.001425x+0.2950 | -0.00143 | y=-0.0009723x+0.1994 | -0. 00097 | y=-0.0004511x+0.09561 | -0. 00045 | **<0.01****** |
|  | IRS | y=-0.001463x+0.3037 | -0.00146 | y=-0.0009933x+0.2042 | -0. 00099 | y=-0.0004682x+0.09952 | -0. 00047 |  |
|  | IRN | y= -0.001429x+0.2903 | -0.00143 | y=-0.0009781+0.1960 | -0. 00098 | y=-0.0004495x+0.099424 | -0. 00045 |  |
|  | IRI | y=-0.001528x+0.2962 | -0.00153 | y=-0.001040x+0.1996 | -0.00104 | y=-0.0004872x+0.09658 | -0. 00049 |  |
|  | IRT | y= -0.001415x+0.2882 | -0.00142 | y=-0.0009559x+0.1942 | -0. 00096 | y=-0.0004562x+0.09383 | -0. 00047 |  |
|  | ORS | y= -0.001647x+0.3161 | -0.00165 | y=-0.001108x+0.2103 | -0. 00111 | y=-0.0005391x+0.1058 | -0. 00054 |  |
|  | ORN | y= -0.001456x+0.2720 | -0.00146 | y=-0.0009781x+0.1818 | -0. 00098 | y=-0.0004768x+0.09021 | -0. 00048 |  |
|  | ORI | y= -0.001687x+0.2979 | -0.00169 | y=-0.001123x+0.1980 | -0. 000112 | y=-0.0005557+0.09963 | -0. 00056 |  |
|  | ORT | y= -0.001448x+0.2692 | -0.00145 | y=-0.0009732x+0.1804 | -0. 00097 | y=-0.0004721x+0.08868 | -0. 00047 |  |
|  | Extramacular | y= -0.001608x+0.2771 | -0.00161 | y=-0.001049x+0.1821 | -0.00105 | y=-0.0005459x+0.09454 | -0. 00055 |  |
| **Pooled ETDRS** | Inner ring | y= -0.001120x+0.2793 | -0.00112 | y=-0.0007560x+0.1878 | -0. 000756 | y=-0.0003627x+0.09144 | -0. 0003627 |  |
|  | Outer ring | y= -0.001210x+0.2729 | -0.00121 | y=-0.0008040x+0.1816 | -0. 000804 | y=-0.0004032x+0.09118 | -0. 0004032 |  |

Abbreviations: IRS- Inner ring superior, IRN- Inner ring Nasal, IRI- Inner ring inferior, IRT-Inner ring temporal, ORS-Outer ring superior, ORN-Outer ring nasal, ORI-Outer ring inferior, ORT-Outer ring Temporal. Significance level: *p<0.05, **p<0.01, *** p<0.001 ,****p<0.0001.
